# The antibacterial and antifungal activity of six essential oils and their cyto/genotoxicity to human HEL 12469 cells

**DOI:** 10.1038/s41598-017-08673-9

**Published:** 2017-08-15

**Authors:** Andrea Puškárová, Mária Bučková, Lucia Kraková, Domenico Pangallo, Katarína Kozics

**Affiliations:** 1 Institute of Molecular Biology, Slovak Academy of Sciences, Dúbravská cesta 21, 84551 Bratislava, Slovakia; 2 Cancer Research Institute, Biomedical Research Center, Slovak Academy of Sciences, Dúbravská cesta 9, 84505 Bratislava, Slovakia

## Abstract

Six essential oils (from oregano, thyme, clove, lavender, clary sage, and arborvitae) exhibited different antibacterial and antifungal properties. Antimicrobial activity was shown against pathogenic (*Escherichia coli*, *Salmonella typhimurium*, *Yersinia enterocolitica*, *Staphylococcus aureus*, *Listeria monocytogenes*, and *Enterococcus faecalis*) and environmental bacteria (*Bacillus cereus*, *Arthrobacter protophormiae*, *Pseudomonas fragi*) and fungi (*Chaetomium globosum, Penicillium chrysogenum*, *Cladosporium cladosporoides*, *Alternaria alternata*, and *Aspergillus fumigatus)*. Oregano, thyme, clove and arborvitae showed very strong antibacterial activity against all tested strains at both full strength and reduced concentrations. These essential oils showed different fungistatic and fungicidal activities when tested by direct application and in the vapor phase. The genotoxic effects of these oils on HEL 12469 human embryo lung cells were evaluated using an alkaline comet assay for the first time, revealing that none of the oils induced significant DNA damage *in vitro* after 24 h. This study provides novel approaches for assessing the antimicrobial potential of essential oils in both direct contact and the vapor phase and also demonstrates the valuable properties of the phenol-free arborvitae oil. These results suggest that all the tested essential oils might be used as broad-spectrum anti-microbial agents for decontaminating an indoor environment.

## Introduction

Essential oils (EOs) are products derived from aromatic plants which contain around 20–60 components at quite different concentrations^1^. Their most common constituents are terpenes, aromatic and aliphatic compounds (especially alcohols, esters, ethers, aldehydes, ketones, lactones, phenols and phenol ethers)^1^.

EOs from *Origanum vulgare* L., *Thymus vulgaris* L., *Salvia sclarea* L., and *Lavandula angustifolia* Mill. belonging to the *Lamiaceae* family have been used for their medicinal properties^1^ for centuries; they possess antibacterial, antifungal^2–5^, antioxidant, anti-inflammatory^6, 7^ and analgesic properties^7^. Clove EO from *Eugenia caryophyllata* L. (*Myrtaceae*) has shown antibacterial, antifungal, anti-oxidant^8^ and anti-inflammatory effects^9^. EO from *Thuja plicata* (*Cupressaceae*) has been tested for antimicrobial^10, 11^ and insecticidal activity^12^.

The antibacterial properties of EOs have been observed in several studies^1, 13, 14^. Most of the studies have examined the direct effect of EOs on a range of microorganisms. For example several Gram-negative and Gram-positive bacteria are sensitive to various EOs^2, 3, 14–16^, showing clear zones on agar assays in which the tested EO inhibits the growth of a particular microorganism. Some studies also determined the minimal inhibition and minimal bactericidal concentrations in liquid medium^11, 17^.

However, EOs can also exist in a potentially highly bioactive vapor phase, and some EOs have shown antimicrobial activity that does not require direct contact with the EO^18–21^. The vapor phase seems especially effective against fungi, and a number of studies have shown that EOs are more effective antifungals in the vapor state than in the liquid^20–22^. One possible explanation for this behavior is that the lipophilic molecules responsible for at least part of the activity might associate in the aqueous phase to form micelles, thereby suppressing their attachment to the organism, whereas the vapor phase allows free attachment^22^. In this situation, the observed antimicrobial activity arising from the easily volatilized components would result from a combination of the direct exposure to the vapor and the indirect exposure mediated by agar medium which absorbed the vapor^23^. Moreover, fungal strains tend to grow more on the agar surface than bacteria, and therefore would be more exposed to the vapor while the bacteria would be more strongly affected by the EO components that accumulated in the substrate.

EOs with biocidal activity were used to develop alternative disinfection strategies for indoor environments or in the food industry, on contaminated surfaces and equipment in food processing environments^15, 24–26^. The ability of some EOs to prevent the formation of *Listeria monocytogenes* ^15^ and *Salmonella enterica* ^26^ biofilm on stainless steel surfaces has previously been demonstrated.

Although EOs were applied in the past to successfully treat a variety of diseases and to preserve health, they have been used more frequently for a greater variety of applications in recent years, including drugs, crop protectants, food additives, aromatherapy, and others. The resulting increase in human exposure as a consequence of this expanded usage therefore requires a careful re-assessment of their toxicity and genotoxicity on the level of mammalian cells^27^. The potential toxic effects of plant extracts, including EOs, on humans should not be underestimated. The mutagenicity of many plant extracts and their possible genotoxicity^28–32^ have been evaluated previously. There are several studies examining the genotoxic properties of EOs^29, 33–35^, but there is not nearly enough information about the potential risk of sensitization when using EOs.

The purpose of this study was to determine the antimicrobial properties of six EOs (*O*. *vulgare*, *T*. *vulgaris*, *S*. *sclarea*, *L*. *angustifolia*, *E*. *caryophyllata* and *T*. *plicata*) against clinical and food-borne bacterial pathogens and as well as several environmental bacterial and fungal strains. The antifungal properties of the vapor phase of these EOs were also investigated. Our *in vitro* trials determined the concentrations of EOs needed to reliably prevent the growth of pathogenic and environmental microorganisms. Finally, in this paper we also report the first *in vitro* results on the cytotoxic and genotoxic activities of these EOs in human embryo lung cells (HEL 12469).

## Results

### Antibacterial activity of essential oils

**Figure 1:**
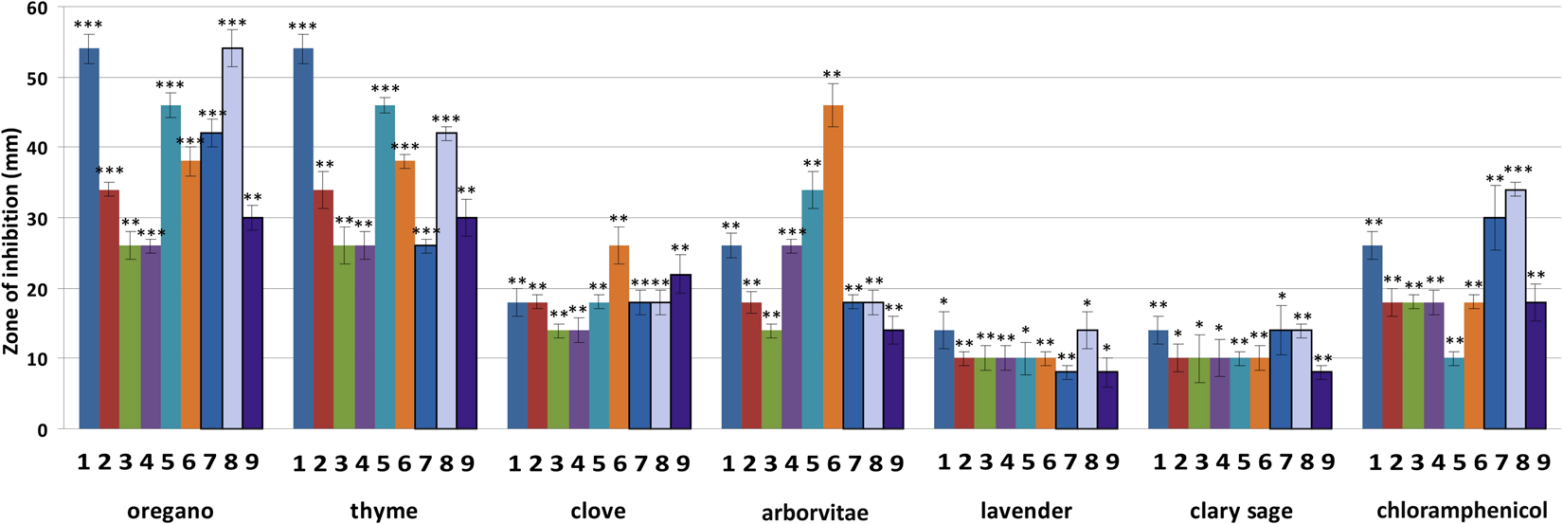
Antimicrobial potential of EOs. Results for the agar diffusion assay performed on the six clinical bacterial strains and three environmental bacterial strains are shown. Chloramphenicol (30 μg/disc) was used as a positive control. Each bar of the chart shows the mean of the inhibitory zone obtained for each EO analyzed (1) *Staphyloccocus aureus*, (2) *Listeria monocytogenes*, (3) *Enterococcus fecalis*, (4) *Escherichia coli*, (5) *Salmonella typhimurium*, (6) *Yersinia enterolitica*, (7) *Bacillus cereus*, (8) *Arthrobacter protophormiae*, (9) *Pseudomonas fragi*. Data are represented by means ± 1 SD of 3 independent experiments. *p < 0.05; **p < 0.01; ***p < 0.001 indicate statistically significant differences compared to the control (Student’s *t*-test).

The *in vitro* antibacterial activity of six EOs against bacterial strains from both clinical and environmental origins (both Gram-positive and Gram-negative bacteria) was assayed using the disc diffusion method by measuring inhibition zone diameters (Fig. 1). All EOs tested showed antibacterial effects based on these inhibition zones (*p < 0.05; **p < 0.01; ***p < 0.001). *Origanum vulgare* (OR) and *Thymus vulgaris* (TY) EOs were extremely effective on all tested bacteria, with inhibition zones ranging from 26–54 mm. The differences in the measured inhibition halos of OR (p = 0.000457), TY (p = 0.000457) and *Lavandula angustifolia* (LA; p = 0.0117) on *Staphylococus aureus* were statistically different from the control. Interestingly, OR and TY produced inhibition halos much larger than those of chloramphenicol, suggesting that they are more active than this antibiotic. *Eugenia caryophyllata* (clove; CL) and *Thuja plicata* (arborvitae; AR) EOs exhibited a lower degree of bacterial growth inhibition than OR and TY, while the greatest inhibition observed was caused by AR against *Yersinia enterocolitica* (p < 0.01). Environmental bacterial strains were much more sensitive to chloramphenicol than clinical strains; no significant difference in susceptibility was found between Gram-negative and Gram-positive bacteria. LA and *Salvia sclarea* (SA) EOs were both less active against all bacteria, with inhibition zones ranging from 8–14 mm.

Preliminary screening revealed that the OR, TY, CL, and AR EOs were the most effective against all tested bacteria; therefore, additional the minimum inhibitory concentration (MIC) and minimum bactericidal concentration (MBC) assays were performed with these four EOs. MIC and MBC assays were performed using a broth microdilution method in 96-well strip tubes covered with strip-caps. The results obtained from these assays are shown in Table 1. These antibacterial assays revealed that OR has a very strong activity (MIC 0.025%, MBC 0.025–0.05%) together with TY (MIC 0.025–0.125%, MBC 0.05–0.125%) while the CL and AR EOs had less antibacterial activity (MIC 0.05–0.125%, MBC 0.125–0.5%). All four EOs inhibited the growth of both clinical and environmental Gram-positive (*S*. *aureus*, *L*. *monocytogenes*, *E*. *faecalis*, *B*. *cereus*, and *A*. *protophormiae)* and Gram-negative bacteria (*E*. *coli*, *S*. *typhimurium*, *Y*. *enterocolitica*, and *P*. *fragi*).

**Table 1:** Minimum inhibitory concentrations (MIC) and minimum bactericidal concentrations (MBC) (% w/v) of oregano, thyme, clove, and arborvitae EOs against tested bacteria.

Microorganisms	Oregano			Thyme		Clove		Arborvitae	
	Origin	MIC	MBC	MIC	MBC	MIC	MBC	MIC	MBC
*Staphyloccocus aureus*	Path	0.025	0.025	0.125	0.125	0.125	0.125	0.25	0.25
*Listeria monocytogenes*	Path	0.025	0.025	0.125	0.125	0.125	0.125	0.25	0.25
*Enterococcus fecalis*	Path	0.025	0.05	0.025	0.05	0.05	0.25	0.125	0.25
*Escherichia coli*	Path	0.025	0.025	0.125	0.125	0.125	0.125	0.125	0.25
*Salmonella typhimurium*	Path	0.025	0.025	0.125	0.125	0.125	0.125	0.125	0.25
*Yersinia enterolitica*	Path	0.025	0.025	0.05	0.05	0.05	0.05	0.05	0.125
*Bacillus cereus*	Env	0.025	0.05	0.05	0.05	0.125	0.5	0.125	0.5
*Arthrobacter protophormiae*	Env	0.025	0.05	0.05	0.125	0.125	0.125	0.125	0.125
*Pseudomonas fragi*	Env	0.025	0.025	0.025	0.05	0.125	0.125	0.125	0.125

Each value is the mean of triplicate assays; Path – Pathogenic; Env – Environmental.

### Antifungal activity of essential oils

**Figure 2:**
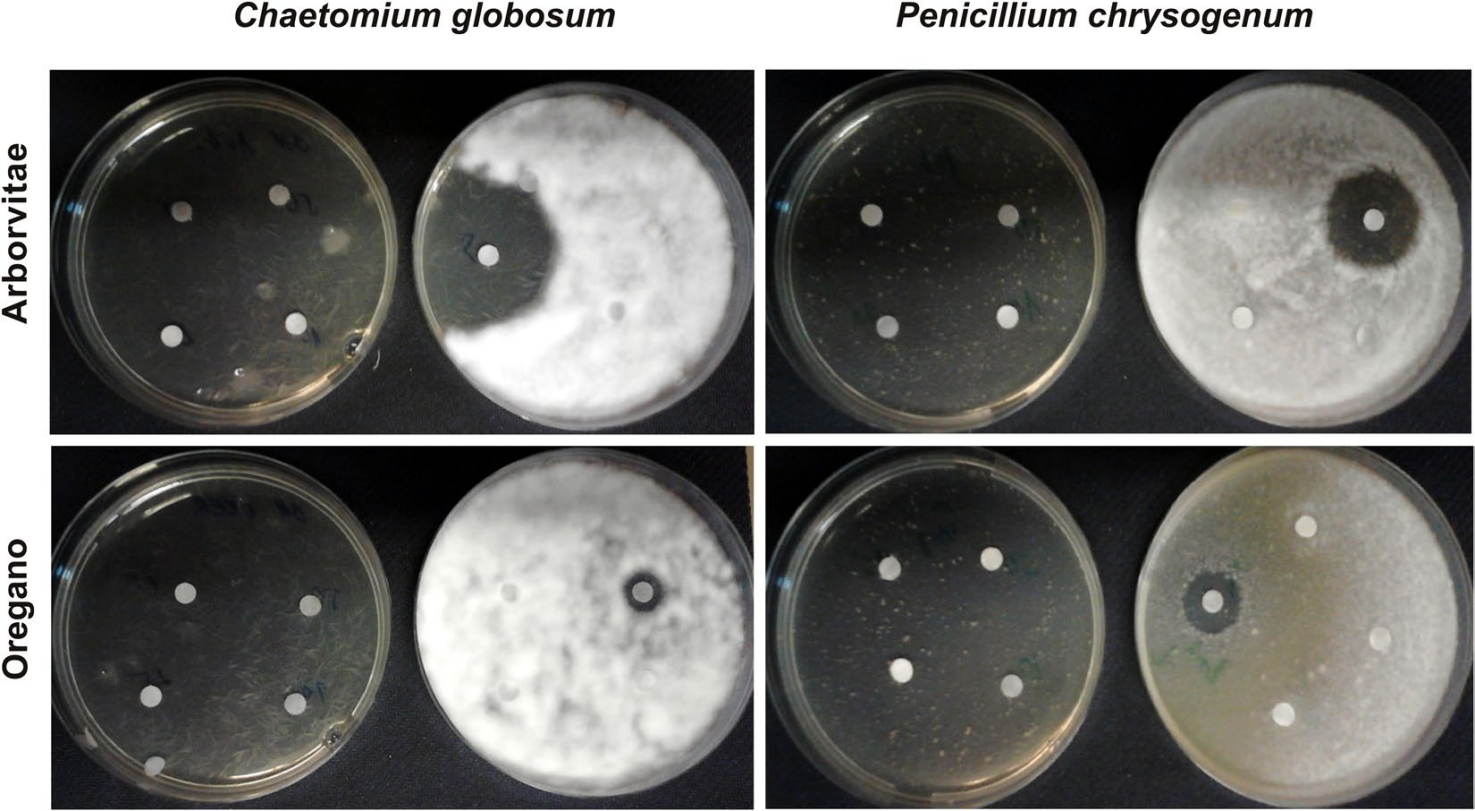
Antifungal activity of arborvitae and oregano EOs against *Chaetomium globosum* and *Penicilium chrysogenum*. The effects of different concentrations (75%, 50%, 25%, 10% – left plates and 5% – right plates) of EOs dissolved in DMSO are shown. The disc-diffusion assay reveals total growth inhibition after treatment with 75%, 50%, 25%, and 10% (w/v) EOs.

**Figure 3:**
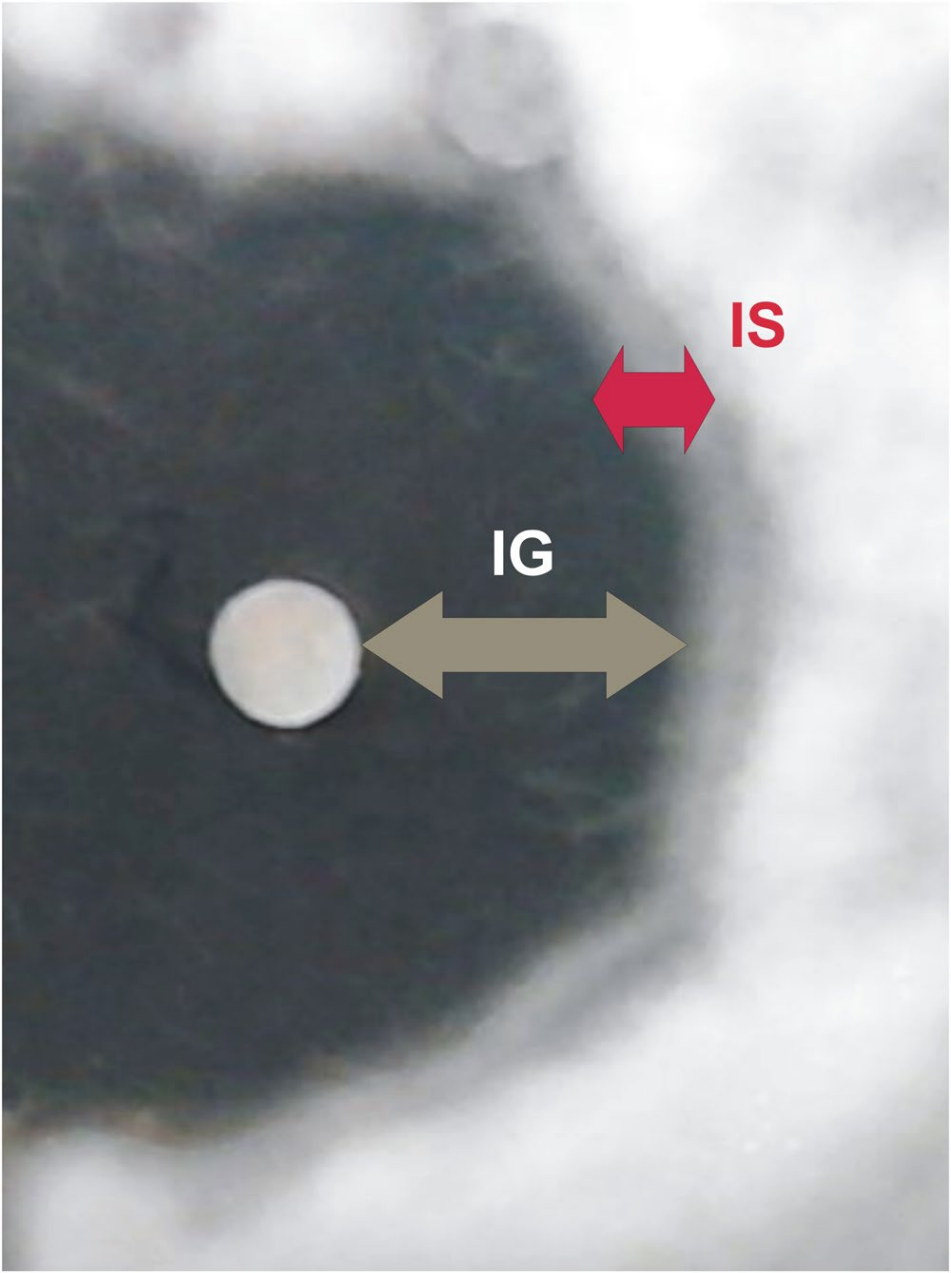
Detailed view of the inhibition of fungal growth and fungal sporulation in *Chaetomium globosum* after treatment with 5% arborvitae EO dissolved in DMSO. Arrows indicate inhibition of fungal growth (gray arrow IG) and inhibition of fungal sporulation (red arrow IS).

A disc diffusion assay was performed to determine the sensitivity of five fungal strains to the six EOs by measuring the inhibition zone diameters (in mm). Our goal was to determine whether the different EOs had similar inhibition effects on several different fungal strains (*Cladosporium cladosporoides*, *Alternaria alternata*, *Aspergillus fumigatus*, *Chaetomium globosum* and *Penicillium chrysogenum*). All tested EOs at concentrations of 75, 50, 25, 10 and 5% (w/v) showed antifungal activity, inhibiting the mycelial growth (Figs 2 and 3). The LA and SA EOs exhibited a lower level of inhibition.

The MIC and minimal fungicidal concentrations (MFC) of the OR, TY, CL, and AR EOs against *Ch*. *globosum, P*. *chrysogenum*, *C*. *cladosporoides*, *A*. *alternata*, and *A*. *fumigatus* are summarized in Table 2. The greatest antifungal activity against all tested strains was exhibited by OR, which had MICs of 0.01% and 0.025% and MFCs of 0.025%, 0.05% and 0.075%. TY EO, despite being efficient against all tested fungal strains, appeared to have no fungicidal activity against *P*. *chrysogenum, C*. *cladosporoides* and *A*. *fumigatus* (Table 2). CL also had no fungicidal activity against *A*. *alternata* and *P*. *chrysogenum*. Overall, OR, AR, TY and CL were effective as fungicidal agents but their efficiency varied from strain to strain (Table 2). The fungicidal effect was confirmed when sub-culturing the tested fungi from the agar dilution assays into fresh malt extract broth (MEB) without EO resulted in no further mycelial growth or resumption of spore germination. LA and SA EOs had no antifungal activity against any tested fungal strain.

**Table 2:** Minimal inhibitory concentrations (MIC) and minimal fungicidal concentrations (MFC) (% w/v) of oregano, thyme, clove, and arborvitae EOs against the tested fungal strains.

Fungi	Oregano		Thyme		Clove		Arborvitae	
	MIC	MFC	MIC	MFC	MIC	MFC	MIC	MFC
*Chaetomium globosum*	0.01	0.025	0.025	0.05	0.025	0.05	0.01	0.025
*Alternaria alternata*	0.01	0.05	0.025	0.05	0.025	ND	0.05	ND
*Penicilium chrysogenum*	0.025	0.075	0.05	ND	0.05	ND	0.025	ND
*Cladosporium cladosporioides*	0.01	0.075	0.025	ND	0.05	0.075	0.01	ND
*Aspergillus fumigatus*	0.025	0.075	0.05	ND	0.025	0.075	0.075	ND

Each value is the mean of triplicate assays, ND – not detected.

### Volatile vapor of essential oils

**Figure 4:**
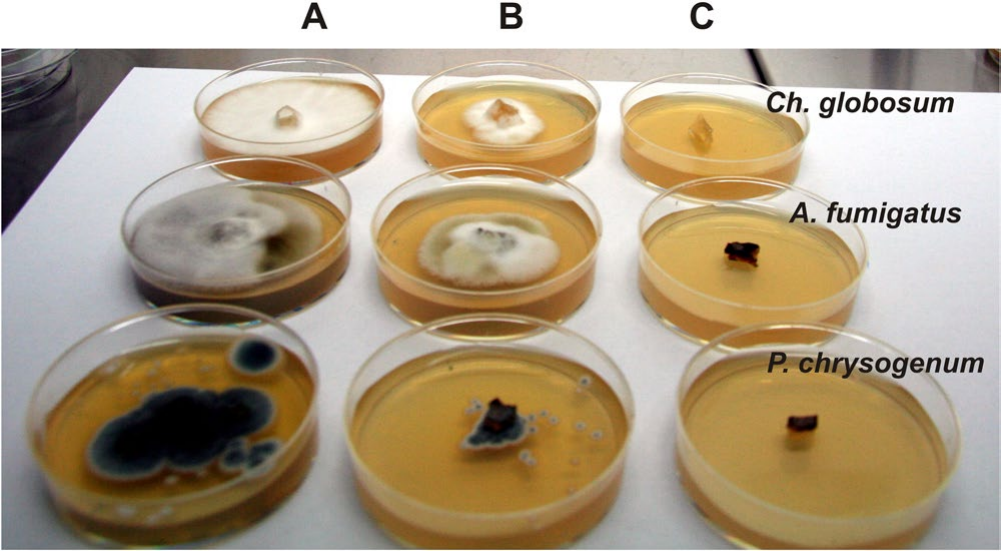
Mycelial growth inhibition of thyme essential oil vapor at different concentrations against *Chaetomium globosum*, *Aspergillus fumigatus* and *Penicillium chrysogenum* on a Malt Extract agar plate. (**A**) control, (**B**) 0.005%, C: 0.075% (w/v) at dose levels of 1 µL/mL air space.

The efficacy of OR, TY, CL, AR, LA, and SA EOs in the vapor phase against *Ch*. *globosum, P*. *chrysogenum*, *C*. *cladosporoides*, *A*. *alternata*, and *A*. *fumigatus* was investigated. The volatile vapor of 0.005% EOs exhibited only a fungistatic effect on the tested fungi while the volatile vapor of 0.075% OR, TY, CL and AR completely inhibited the mycelial growth of all tested fungal strains (Fig. 4) and were also revealed to have a fungicidal effect after the re-inoculation of inhibited fungal mycelial plugs into fresh malt extract agar (MEA) and fresh MEB. Exceptionally, however, *P*. *chrysogenum* and *A*. *fumigatus* treated with CL volatile vapor (0.075%) continued to grow in fresh MEB after re-inoculation, meaning that CL had only a fungistatic effect on these strains.

The volatile vapor of LA and SA at 0.075% concentration completely inhibited the growth of all tested fungi except *A*. *alternata*, but had no fungicidal properties against any of them. The vapor phase of TY and AR were more effective against *P*. *chrysogenum*, *C*. *cladosporoides* and *A*. *fumigatus* than in the liquid phase.

### Cytotoxic and DNA-damaging effects of essential oils

**Figure 5:**
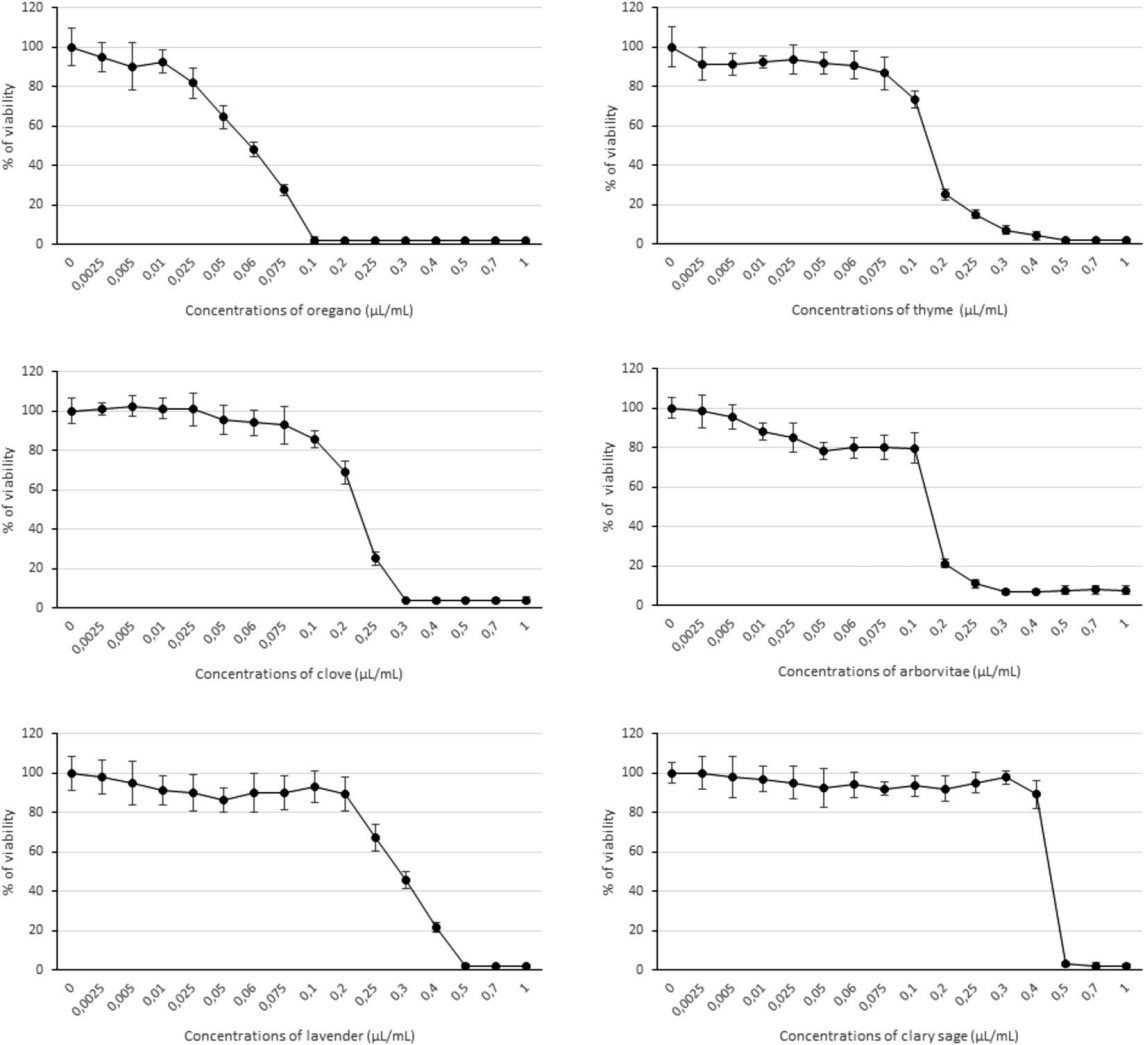
Cytotoxicity or viability of human HEL 12469 cells. The effects of a 24 h treatment of different concentrations of EOs (0.0025–1.0 µL/mL) are shown. Data are represented means ± 1 SD of 3 independent experiments.

The MTT (3-(4,5-dimethylthiazol-2-yl)-2,5-diphenyltetrazolium bromide) assay was used to determine the cytotoxic effects on HEL 12469 cells of a 24 h exposure of different concentrations of EOs (0.0025–1.0 µL/mL). Figure 5 summarizes the results: IC_50_ values (the median inhibitory concentrations that cause approximately 50% cell death) were 0.058 µL/mL for OR, 0.15 µL/mL for AR and TY, 0.23 µL/mL for CL, 0.28 µL/mL for LA, and 0.45 µL/mL for SA; IC_20_ values (the median inhibitory concentrations that cause approximately 20% cell death) were 0.026 µL/mL for OR, 0.10 µL/mL for AR, 0.085 µL/mL for TY, 0.13 µL/mL for CL, 0.23 µL/mL for LA, and 0.41 µL/mL for SA.

**Figure 6:**
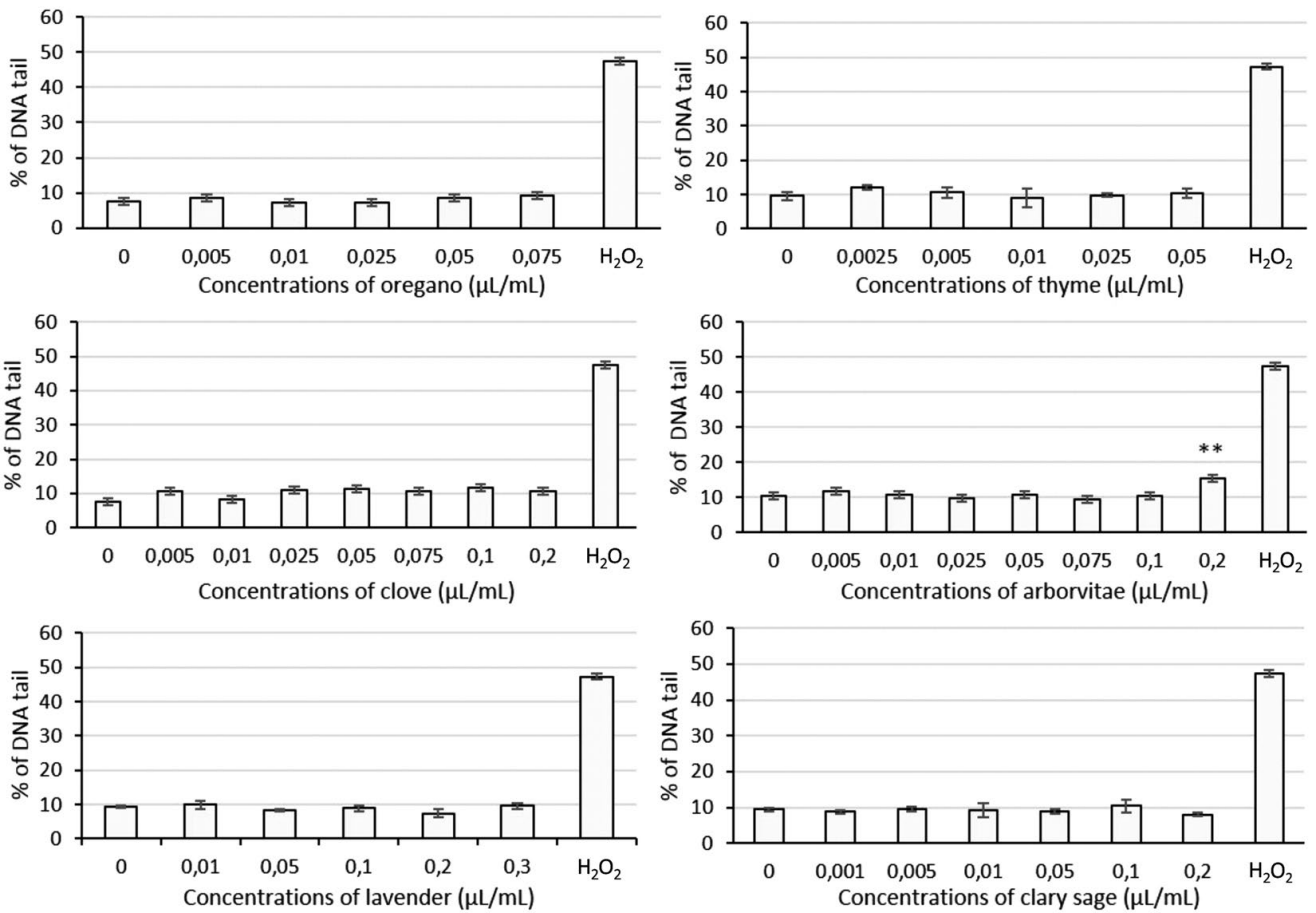
The levels of DNA single-strand breaks in HEL 12469 cells pre-treated with EOs for 24 h. As positive control, hydrogen peroxide (300 μM) was used. Data are represented means ± 1 SD of 3 independent experiments. *p < 0.05; **p < 0.01; ***p < 0.001 indicate statistically significant differences compared to untreated control cells (ANOVA test).

Further studies examined the genotoxic effects of these EOs, which were assessed at IC~_10–40_. Single-cell gel electrophoresis (SCGE; also known as comet assay) was used to determine the level of DNA single-strand breaks in HEL 12469 cells. Only one of the EOs, 0.2 µl/ml of AR, induced a significantly different level of DNA breaks than those observed in the untreated control cells, (**p < 0.01) (Fig. 6).

## Discussion

The antimicrobial efficacy of a given EO depends on its chemical composition, perhaps especially its phenolic components^1, 14^. In our study, therefore, we selected the EOs which are well-known for its high content phenols (OR, TY, CL), EOs with lower phenol content (LA and SA), and a phenol-free EO (AR)^11, 36, 37^. This last was chosen based on previous studies on the susceptibility of various bacteria and fungi to cedar leaf EO^36^ and on our own preliminary experiments (data not shown). This particular AR EO was obtained from heart-wood and contained mainly tropolones^37^.

The pathogenic bacteria selected for this study were chosen based on previous findings on the power of some EOs to inhibit some human pathogens^2, 4, 17, 38^. The environmental bacterial and fungal strains were chosen to be representative of airborne contaminants which our group has isolated from indoor environments (unpublished data).

The measured inhibition halos of OR, TY, CL, AR, LA, SA indicated that all of EOs are effective against bacteria. The OR and TY used in this study were even more effective than the antibiotic chloramphenicol. Our results are in accordance with a previous study showing that the inhibitory halos produced by the EOs of *Eugenia caryophyllata*, *Origanum vulgare* and *Thymus vulgaris* were larger than those produced by ciprofloxacin^38^.

The low antibacterial activity of the LA and SA EOs may be due to the relatively low phenol content of these EOs: their main components are, respectively, alcohols^39^ and esters^40^. This is consistent with another study on the antimicrobial efficiency of the EO^16^ from *Salvia officinalis*, which reported a very low antibacterial activity for 1,8-cineole against *S*. *aureus*, *B*. *subtilis*, and *E*. *coli*. In the present study, CL EO, which is known for its high eugenol^41^ content (a phenolic compound) was not found to be the most active EO against the microorganisms tested in the disc-diffusion assay. It is possible that the sample we used had a lower concentration of the relevant compounds; it has previously been shown that the EOs of plants belonging to the same species, but collected from different places can exhibit different antimicrobial activity^42^.

It should be noted that the disc-diffusion method is limited by the hydrophobic nature of most EOs, which prevents their uniform diffusion through the agar medium. Therefore, most researchers prefer liquid medium methods^43^. The EOs of OR, TY, CL and AR exhibited strong antimicrobial activity against all microorganisms in liquid medium, as it has been previously described^3, 16^. It is also quite interesting that no bacterial strain tested was resistant to any of the EOs studied.

Some studies have reported that EOs tend to act more strongly on Gram-positive than Gram-negative bacteria^2–4, 44^, presumably due to differences in cell wall composition^44^. There is no general rule with respect to Gram sensitivity: the literature reports many conflicting studies showing that some Gram-negative strains are more sensitive than some Gram-positive ones to certain EOs^21, 36, 38^. For example, Preuss *et al*.^45^ found that origanum EO is lethal to *E*. *coli* and *Klebsiella pneumoniae*. *Origanum syriacum* L., *Thymus syriacus* Boiss. and *Syzygium aromaticum* L. EOs were effective against the Gram-negative bacteria *E*. *coli* O157:H7, *Y*. *enterocolitica* O9, *Proteus* spp., and *K*. *pneumoniae* ^46^. Our results have also shown that some Gram-negative bacteria (*E*. *coli*, *S*. *typhimurium*, *Y*. *enterocolitica*, and *P*. *fragi)* are sensitive to OR, TY, CL, AR.

Antifungal activity of EOs was determined by direct contact assay and also we tested their antifungal properties in the vapor phase. In a previous study, MICs of thyme red, clove, sage and lavender, for aspergilli and penicillin, ranged from 0.125% to 1% after 3 and 7 days^19^. Our results revealed that all tested fungal strains showed higher susceptibility (MICs of OR, TY, CL and AR EOs ranged from 0.01% - 0.075%). The volatile components of OR, TY, CL and AR showed fungicidal activity while the LA and SA vapors demonstrated fungistatic activity. These results are very different from the direct contact assay, where LA and SA were unsuccessful in inhibiting the growth of the tested strains. There is growing evidence that EOs in the vapor phase are more effective against fungi than in the liquid phase^18–22, 47^. Thyme EOs vapours have been shown to be effective against *Aspergillus* sp. and *Penicillium* sp.^19^. The high antifungal activity of vapour evidenced in our study is in accordance with previous finding^48^, which showed that thyme and clove oils were more effective in vapour state against *A*. *flavus*.

AR vapors displayed comparable results to OR, TY and CL in spite of its lack of phenolic compounds and its high concentration of monoterpens, which are normally considered to be less effective antimicrobial substances^14^. According to^14^, antimicrobial activity can generally be classified in the following order: phenols > aldehydes > ketones > alcohols > esters > hydrocarbons. The link between the most abundant constituent type and the antimicrobial activity is somewhat variable; for example, Inouye *et al*.^22^ reported that alcohol-containing EOs are more active than ketone-containing EOs against *Trichophyton mentagrophytes*.

Most EOs are safe and free of adverse side effects when used properly^49^. The most important safety factor for EOs is their dosage. EOs have shown antitumor activity both *in vitro* and *in vivo* and low toxicity^50, 51^. On the other hand, it has also been shown that high concentrations of some EOs contributed to harmful changes in the body^52^.

We have demonstrated the EOs do have cytotoxic effect, but only at higher concentrations on HEL 12469 human embryonic lung cells under *in vitro* conditions. The effect of a 24 h treatment with a given EO on the viability of HEL cells was dependent on its concentration. IC_50_ values declined in the order SA > LA > AR = TY > CL > OR. In other cell lines (human hepatocarcinoma cell line HepG2, human keratinocytes HaCaT, human melanoma cell line HMB-2), the cytotoxic effect of LA EO was detected at a slightly higher concentration ~ IC_50_-0.4 µL/mL, which might be explained by differences in cell sensitivity^53^. Finally, LA at high concentration was genotoxic to peripheral human lymphocytes^54^ and to human monocyte THP-1 cells^55^.

In our experiments the EC_50_ value for all EOs was >20 µg/mL (for example: IC_50_ SA - 0.45 µl / ml = 0.351 mg/ml) indicating that none of them were cytotoxic based on the criteria set by the National Cancer Institute^56^, which state that only natural substances with EC_50_ < 20 µg/mL are considered cytotoxic.

The level of DNA single-strand breaks induced in HEL 12469 cells by these EOs was determined using a Comet assay. Treatment with most EOs alone did not induce any significant increase in DNA strand breaks over the untreated control cells; the single exception was the highest concentration of AR EO examined (0.2 µL/mL). Similarly, it was recently shown that plant extracts of *S*. *officinalis* and *T*. *vulgaris* did not induce DNA damage in HepG2 cells or primary rat hepatocytes^57, 58^.

## Conclusions

This study provides a broad range of information about the biological activities of EOs. It determined the biocidal efficiency of six EOs (from OR, TY, CL, AR, LA and SA) against five different fungal and nine different bacterial strains. In order to verify the potential risk of EOs to human cells, the cytotoxicity and genotoxicity of each of these EOs on human HEL lung cells was assessed for the first time. Of the six EOs studied, OR, TY, CL, and AR were highly effective against all bacterial strains tested. LA and SA exhibited no antifungal activity by direct contact, but did show a fungistatic effect in the vapor phase. OR, TY, and AR exhibited important fungicidal activity against all strains tested; CL showed fungicidal activity against most strains, but only a fungistatic effect on *P*. *chrysogenum* and *A*. *fumigatus*.

The assayed EOs are not considered cytotoxic as judged by the criteria set by the National Cancer Institute and appeared not to damage the DNA of HEL cells.

The data reported in this study show that EOs might provide an alternative way to fight microbial contamination and that they can be considered safe for humans at relatively low concentrations. Generally, it is possible to recommend the use of EOs for various environmental disinfection strategies, but only after accurate *in vitro* trials, such as those described in this investigation.

## Materials and Methods

### Essential oils

The commercially available EOs used in this work were OR from *O*. *vulgare* L., TY from *T*. *vulgaris* L., CL from *E*. *caryophyllata* L., LA from *L*. *angustifolia* Mill., SA from *S*. *sclarea* L., and AR from *T*. *plicata* Donn. (all from doTERRA, Pleasant Grove, USA). A GC/MS analysis was provided by the producer, who guaranteed the chemical composition of each EO. The EOs were stored in amber glass vials and sampled using sterile pipet tips to minimize contamination and oxygen exposure. Since the EOs varied in density, each of the EOs was weighed to determine the volume that comprised 10 mg. This amount was used in testing as the full-strength (100%) concentration and was then serially diluted in dimethyl sulfoxide (DMSO; Sigma-Aldrich Co., USA).

### Microbial strains and growth conditions

The EO antimicrobial activities were investigated against different clinical and food-borne bacterial pathogens: *S*. *aureus* (FRIC 418), *L*. *monocytogenes* (FRIC 270), *E*. *faecalis* (FRIC 282); *E*. *coli* (FRIC 375), *S*. *typhimurium* (FRIC 305), and *Y*. *enterocolitica* (FRIC 30); environmental bacterial strains from our own collection were also examined, including *B*. *cereus*, *P*. *fragi*, and *A*. *protophormiae*.

The fungal strains used in this study (*Ch*. *globosum*, *P*. *chrysogenum*, *C*. *cladosporioides*, *A*. *alternata*, *A*. *fumigatus*) were air-borne isolates from our laboratory collection. The bacterial strains were kept frozen in stock cultures at −80 °C in cryovials, and the fungal cultures were stored at 4 °C and subcultured once a month. Prior to the inoculation of the strains with EOs, the bacteria were grown at 28 or 37 °C (depending on the type of microorganism) for 12–18 h on Luria–Bertani agar (LBA) for environmental bacteria or Mueller Hinton agar (MHA) for pathogenic bacteria. The fungal strains were grown at 26 °C on Malt Extract Agar (MEA).

### Cell culture

HEL 12469 human embryo lung cells (Human embryonic lung fibroblast; ECACC 94101201), were cultivated in Eagle’s Minimum Essential Medium (MEM) supplemented with 10% fetal calf serum (FCS), 1% non-essential amino acids and antibiotics (streptomycin 50 μg/mL and penicillin 50 U/mL)^50^. Cell lines were cultured in a humidified atmosphere of 5% CO_2_ at 37 °C. The chemicals and media used for cell cultivation were purchased from Gibco BRL (Paisley, UK).

### Screening for antibacterial activity

A disc-diffusion assay was used to determine the growth inhibition of bacteria by EOs. A single colony from an overnight bacterial culture plate was seeded into 5 mL of an appropriate pre-warmed growth medium broth (LBB or MHB). Culture tubes were shaken at 300 rpm and 37 °C until the 600 nm absorbance of the growth solution was greater than 1.0. Using a sterile swab, cultures were spread evenly onto pre-warmed 37 °C agar plates. Sterile filter paper discs (6 mm Ø Whatman No.1) were gently pressed onto the surface of the agar plates, and EOs were then pipetted onto the discs. Each EO was tested at 100% strength, and at various dilutions (5, 10, 25, 50, and 75%) in DMSO. A pure DMSO control was included with each test to ensure that microbial growth was not inhibited by DMSO itself. Chloramphenicol (30 μg/disc; Sigma-Aldrich, USA) was used as a positive control. Plates were then inverted and incubated for approximately 24 h at 37 °C and the diameter of the inhibition zones was measured in mm, including the diameter of the disc. The sensitivity was classified according to Ponce *et al*.^59^ as follows: not sensitive for a diameter less than 8 mm, sensitive for a diameter of 9–14 mm, very sensitive for a diameter of 15–19 mm, and extremely sensitive for a diameter larger than 20 mm. Each test was performed in three replicates.

### Evaluation of MIC and MBC in liquid medium

The MIC and MBC of each EO was determined using a broth microdilution method in 96-well strip tubes with transparent strip-caps according to Poaty *et al*.^17^ with modification. Bacterial suspensions were adjusted to a final concentration of 10^6^ CFU/mL in MHB. One hundred microliters of MHB containing 5% DMSO was distributed into the wells of the micro titer plates. EOs (10 µL) were added to these wells at a range of concentrations, from stock solutions 0.05, 0.10, 0.50, 1.0, 2.5, 5.0, and 10% (w/v). For each dilution, the same volume as the full-strength sample was added. One hundred microliters of bacterial suspension was finally added to each. The plates were incubated at 37 °C for 24 h. MIC was determined after adding 40 µL of 0.2 mg/mL ρ-iodonitrotetrazolium violet (INT; Sigma-Aldrich, USA), followed by incubation at 37 °C for 30 min. MIC was determined as the lowest concentration of EO that inhibited visible growth of the tested microorganism. Growth of bacterial cells in each of the wells was verified by color change. When bacterial growth occurred (absence of inhibition), the INT changed from clear to purple. Wells with DMSO alone were used as controls. MBC is the lowest concentration of EO that results in microbial death. It was determined by subculturing from wells that exhibited no color change to sterile MHA plates that do not contain the test EO. The plates were then incubated at 37 °C for 24 h.

### Screening for antifungal activity

Fungal suspensions were prepared according to De Lira Mota *et al*.^60^ by washing the surface of the MEA slant culture with 5 mL of sterile saline and shaking the suspensions for 5 min. The resulting mixture of sporangiospores and hyphal fragments was withdrawn and transferred to a sterile tube. After heavy particles were allowed to settle for 3–5 min, the upper suspension was collected and vortexed for 15 s. Final conidia suspensions were adjusted using a Neubauer’s chamber to 10^6^ conidia per mL. 300 µL of each fungal suspension were applied to MEA plates. Filter paper discs (6 mm Ø Whatman No.1) were placed on the agar surface of the Petri dishes and each EO, dissolved in DMSO at different concentrations (75, 50, 25, 10, 5%) was individually added. For each dilution, the same volume as the full-strength sample was placed on the sterile disc. Discs impregnated with 10 µL of DMSO, nystatin (50 µg/mL) and cycloheximide (50 µg/mL) (all Sigma-Aldrich, USA) were used as controls. Petri dishes were incubated at 26 °C for 5 days. Inhibition zone diameters were measured in mm. An inhibition zone larger than 1 mm was taken to indicate a positive effect.

### Determination of fungistatic and fungicidal activities

The procedure reported by Thompson^61^ was used to determine whether a given EO possessed only a fungistatic effect or if it also had fungicidal activity. Different concentrations (10 µL) of each stock EO solution, ranging from 0.50, 1.0, 2.5, 5.0, 10, 25, 50 and 75% (w/v), were prepared by mixing various quantities of a given EO in DMSO (v/v) with 10 mL molten MEA; this mixture was then poured into sterile Petri dishes. The center of each solidified medium was inoculated upside down with 6-mm square mycelial plugs cut from the periphery of 7-day-old cultures. Positive controls were simultaneously run with DMSO and without EO. After incubation of the plates for 7 days at 26 °C, those fungal plugs that did not show any growth were transferred to fresh MEA plates without EO for an additional 7 days at 26 °C to determine which concentration of each EO had a fungicidal effect. The lowest concentration of each EO that completely prevented visible fungal growth and allowed a revival of fungal growth during the transfer experiment was considered the MIC for that EO. This effect was identified as fungistatic. The concentration unfavorable for growth revival during the transfer experiment was taken as the MFC and this effect was identified as fungicidal. Seven days after reinoculation, the inhibited fungal mycelial plugs were once again reinoculated into fresh MEB without EO to see if their growth revived. Microscopic observations were carried out to investigate fungal cell growth after 5 days incubation at 26 °C. No growth was taken to confirm again the fungicidal activity and also to suggest a possible sporocidal effect.

### Antifungal activity of vapor phase of essential oils

In order to determine the fungistatic or fungicidal activity of volatized EOs, 6 mm squares of growing fungal mycelia were taken from the margin of the active growth area of fungal colonies and placed onto MEA plates. EOs from stock solutions (5% and 75%) at dose levels of 1 µL/1 mL air space were placed on the inner surface of the Petri dish lid; controls with DMSO and without EO were also prepared. The plates were sealed with parafilm to prevent vapor leakage and were incubated inverted for 7 days at 26 °C. The radial mycelial growth of the fungus was then checked.

Transfer experiments for determining the fungistatic or fungicidal activity of EO vapors were carried out by replacing the Petri dish lid with a new, untreated one and incubating in an inverted orientation for an additional 7 days at 26 °C. The effect was identified as fungistatic if growth was observed after the new incubation period, and fungicidal if no growth was observed^62^. The effect was also confirmed by reinoculating the inhibited fungal mycelial plugs into fresh MEB without EO.

### Cytotoxicity of essential oils

Exponentially growing HEL 12469 cells cultured in complete MEM were seeded onto 96 well plates (density of 2 × 10^4^ cells/well) and later incubated in the presence or absence (negative control) of 0.0025–1.0 µL/mL EO for 24 h to test for cytotoxicity using the MTT assay^58^. The MTT test is a colorimetric method for measuring the activity of the mitochondrial enzymes that reduce MTT, a yellow tetrazole, to purple formazan. This reduction takes place only when reductase enzymes are active, and therefore conversion is often used as a measure of viable (living) cells. In our experiments, after incubation with EO, HEL 12469 cells were washed with fresh MEM and 100 μL of complete MEM medium and 50 μL of 1 mg/mL MTT solution was added followed by a 4 h incubation. The MTT solution was then replaced with 100 μL of DMSO and the plates were placed on an orbital shaker for 30 min to completely dissolve the formazan crystals. At least 4 parallel wells were used for each sample. Absorbance at 540 nm was measured using an xMark™ Microplate Spectrophotometer (Bio-Rad Laboratories, Inc.) and the background absorbance at 690 nm was subtracted.

### Genotoxicity of essential oils

HEL 12469 cells were seeded into a series of Petri dishes (1 × 10^6^ cells, Ø = 60 mm) and cultured in MEM. Cells were then exposed to different EO concentrations (0.0025–1.0 µL/mL) for 24 h; cells with no treatment were used as an intact control. After the treatment, the cells were washed, trypsinized, re-suspended in a fresh culture medium and the level of DNA lesions was detected using the single cell gel electrophoresis (SCGE), also known as comet assay (alkaline). The procedure of Singh *et al*.^63^ was used with minor modifications suggested by Slameňová *et al*.^64^. In brief, 2 × 10^4^ treated and control HEL 12469 cells were embedded in 0.75% low melting point (LMP) agarose. This cell suspension was spread as a single layer on a base layer of 1% normal melting point (NMP) agarose in PBS on microscopic slides and covered with cover slips. As positive control, hydrogen peroxide (300 μM) was used. After solidification of the gel, the cover slips were removed and placed in lysis solution (2.5 M NaCl, 100 mM Na_2_EDTA, 10 mM Tris-HCl, pH 10 and 1% Triton X-100, at 4 °C) for 1 h to remove cellular proteins and membranes. The slides were transferred to an electrophoresis box and immersed in an alkaline solution (300 mM NaOH, 1 mM Na_2_EDTA, pH > 13). After 40 min unwinding time, a voltage of 25 V (0.6 V/cm) was applied for 30 min at 4 °C. The slides were then neutralized with 3 × 5 min washes with Tris-HCl (0.4 M, pH 7.4), and stained with ethidium bromide (EtBr, 5 µg/mL; Sigma Chemical Company, St. Louis, MO). EtBr-stained nucleoides were examined with a Zeiss Imager Z2 fluorescence microscope with computerized image analysis (Metafer 3.6, Meta Systems GmbH, Altlussheim, Germany). The percentage of DNA in the tail was used as a parameter for estimating the number of DNA strand breaks. One hundred comets were scored for each sample in one electrophoresis run.

### Statistical analysis

The data are given as means of 3 to 5 experiments ± one standard deviation (SD). The differences between the given groups were tested for statistical significance using Student’s *t*-test (*p < 0.05; **p < 0.01; ***p < 0.001)^50^. Because the antibacterial activity datasets were normally distributed, the independent samples *t*-test was performed to test for significant differences between groups. Differences between more than two groups were assessed by one way analysis of variance (ANOVA) followed by the Bonferroni test if equal variances were assumed or Tamhane’s test if equal variances were not assumed^50^. Differences with *p* < 0.05 are considered to be statistically significant.
